# Proteomic Characterization of Plasmid pLA1 for Biodegradation of Polycyclic Aromatic Hydrocarbons in the Marine Bacterium, *Novosphingobium pentaromativorans* US6-1

**DOI:** 10.1371/journal.pone.0090812

**Published:** 2014-03-07

**Authors:** Sung Ho Yun, Chi-Won Choi, Sang-Yeop Lee, Yeol Gyun Lee, Joseph Kwon, Sun Hee Leem, Young Ho Chung, Hyung-Yeel Kahng, Sang Jin Kim, Kae Kyoung Kwon, Seung Il Kim

**Affiliations:** 1 Division of Life Science, Korea Basic Science Institute, Daejeon, Republic of Korea; 2 Department of Biology, Dong-A University, Busan, Republic of Korea; 3 Department of Environmental Education, Sunchon National University, Sunchon, Republic of Korea; 4 Korea Institute of Ocean Science & Technology, Ansan, Republic of Korea; 5 Department of Bio-Analytical Science, University of Science and Technology (UST), Daejeon, Republic of Korea; Cincinnati Childrens Hospital Medical Center, United States of America

## Abstract

*Novosphingobium pentaromativorans* US6-1 is a halophilic marine bacterium able to degrade polycyclic aromatic hydrocarbons (PAHs). Genome sequence analysis revealed that the large plasmid pLA1 present in *N. pentaromativorans* US6-1 consists of 199 ORFs and possess putative biodegradation genes that may be involved in PAH degradation. 1-DE/LC-MS/MS analysis of *N. pentaromativorans* US6-1 cultured in the presence of different PAHs and monocyclic aromatic hydrocarbons (MAHs) identified approximately 1,000 and 1,400 proteins, respectively. Up-regulated biodegradation enzymes, including those belonging to pLA1, were quantitatively compared. Among the PAHs, phenanthrene induced the strongest up-regulation of extradiol cleavage pathway enzymes such as ring-hydroxylating dioxygenase, putative biphenyl-2,3-diol 1,2-dioxygenase, and catechol 2,3-dioxygenase in pLA1. These enzymes lead the initial step of the lower catabolic pathway of aromatic hydrocarbons through the extradiol cleavage pathway and participate in the attack of PAH ring cleavage, respectively. However, *N. pentaromativorans* US6-1 cultured with *p*-hydroxybenzoate induced activation of another extradiol cleavage pathway, the protocatechuate 4,5-dioxygenase pathway, that originated from chromosomal genes. These results suggest that *N. pentaromativorans* US6-1 utilizes two different extradiol pathways and plasmid pLA1 might play a key role in the biodegradation of PAH in *N. pentaromativorans* US6-1.

## Introduction

Environmental contamination by polycyclic aromatic hydrocarbons (PAHs) is a serious problem due to their toxic, carcinogenic and recalcitrant properties [1], and hence their biodegradation is an important process crucial for bioremediation, and understanding the breakdown pathways is an important part of developing clean-up technology. High-resolution analytical chemistry for metabolomics and high-throughput sequencing for genomics are essential for resolving PAH biodegradation pathways. Recently, high-throughput proteomic approaches and integrated omics technologies have become important tools in the discovery of related proteins and enzymes [2], [3]. For example, metabolite analysis and proteogenomic methods were recently used to fully understand the biodegradation of pyrene in *Mycobacterium vanbaalenii* PYR-1 [4]–[7]. Proteomic studies have also been conducted on the PAH-degrading *Sphingomonas* sp. CHY-1 and *Mycobacterium* sp. KMS [8], [9].

*Novosphingobium pentaromativorans* US6-1 is a Gram negative halophilic marine bacterium able to utilize several PAHs, including phenanthrene, pyrene, and benzo[a]pyrene, as sole carbon and energy sources [10]. Genome sequencing of *N. pentaromativorans* US6-1 has been recently completed and the genome database is accessible from the public NCBI database [11]. *N. pentaromativorans* US6-1 contains two plasmids, pLA1 and pLA2. Large plasmid pLA1 possesses clustered putative aromatic compound degradation genes. The purpose of this study was to elucidate the PAH biodegradation pathways active in *N. pentaromativorans* US6-1. Proteomic analysis of *N. pentaromativorans* US6-1 cultured in the presence of different PAHs was performed to identify biodegradation-related proteins and revealed the induction of low-molecular-weight (LMW) aromatic-hydrocarbon degrading genes located in pLA1. Importantly, this strain uses a plasmid-born extradiol cleavage pathway (catechol-2,3 dioxygenase pathway) for the degradation of both PAHs and MAHs. In this study, we report on the role of plasmid pLA1 in PAH biodegradation and the physiological characterization of *N. pentaromativorans* US6-1 using proteomic approach. Genomic studies on the biodegradation plasmids from several stains have been conducted previously [12]. However, proteomic characterization of these extrachromosomal genetic elements has yet to be performed. This study reports the proteomic analysis of a biodegradation plasmid.

## Materials and Methods

### Bacteria cultivation and sample preparation

*N. pentaromativorans* US6-1 was cultured according to a method described previously [10]. A starter culture of bacteria was prepared by growing cells in marine broth (MB) at 30°Cto an optical density at 600 nm (OD_600_) of 0.8. Bacteria were harvested aseptically and equal amounts of bacteria (culture of 500 ml) were added to fresh Bushnell-Hass broth (incorporating 30 g NaCl/L) (BD, USA) containing phenanthrene (50 ppm), pyrene (50 ppm), benzo(a)pyrene (50 ppm), benzoate (50 ppm), and *p*-hydroxybenzoate (50 ppm). OD_600_ values of all cultures were checked at every six hours until 48 hours using spectrophotometer (Beckman coulter proteome Lab Du 800, USA). Bacteria were harvested after 12 or 36 h before suspending in 20 mM Tris-HCl buffer (pH 8.0) and then disrupting twice in a French pressure cell (SLM AMINCO, Urbana, IL, USA) at 20,000 lb/in^2^. Supernatants (crude cell extracts) were collected by centrifugation (15,000×*g*, 45 min) and subjected to oxygenase activity assay and proteomic analysis. Protein concentrations were determined by the Bradford method [13]. Enzyme activity assay and proteome analysis was conducted on the basis of same protein amount.

### Dioxygenase activity assay

The activities of catechol 1,2-dioxygenase (CD1,2), catechol 2,3-dioxygenase (CD2,3), protocatechuate 3,4-dioxygenase (PCD3,4), and protocatechuate 4,5-dioxygenase (PCD4,5) were determined using a UV spectrometer (Beckman Coulter Proteome Lab DU800, USA), as reported previously [14]. The activities of CD1,2 and CD2,3 were assayed by monitoring increases in concentration of *cis*, *cis*-muconate at A_260_ and 2-hydroxymuconic semialdehyde at A_375_, respectively. Activities of PCD3,4 was determined by monitoring the increase in concentration of β-carboxymuconate at A_290_ (absorbance decreased as β-carboxymuconate concentrations increased) and PCD4,5 activity was measured through the increase in 2-hydroxy-4-carboxy-muconic semialdehyde at A_410_, respectively. For each assay, one unit (U) of enzyme activity is defined as the amount of enzyme producing 1 mmol of product per min. Activity assay of each sample was conducted at least three times for each sample.

### One-dimensional gel electrophoresis (1-DE) and in-gel digestion

SDS-PAGE and in-gel digestion was performed as reported previously [15]. After electrophoresis and Coomassie blue staining, 1D-gels were divided into 10 fractions according to molecular weight. Sliced gels containing fractionated protein samples were digested with trypsin (Promega, Madison, WI, USA) for 16 h at 37°C after reduction with 10 mM dithiothreitol (DTT) and alkylation of cysteines with 55 mM iodoacetamide. The digested peptides were recovered with extraction solution (50 mmol/L ammonium bicarbonate, 50% acetonitrile, and 5% trifluoroacetic acid). The extracted tryptic peptides were dissolved in 0.5% trifluoroacetic acid prior to further fractionation by LC-MS/MS analysis.

### LC MS/MS analysis

LC MS/MS analysis was performed according to a modified version of a previously published method [16]. Tryptic peptide samples were loaded onto a 2G-V/V trap column (Waters, USA) for the enrichment of peptides and the removal of chemical contaminants. Concentrated tryptic peptides were eluted from the column and directed onto a 10 cm ×75 µm i.d. C18 reverse phase column (PROXEON, Denmark) at a flow rate of 300 nl/min. Peptides were eluted using a gradient of 0−65% acetonitrile for 80 min. All MS and MS/MS spectra were acquired in a data-dependent mode using a LTQ-Velos ESI Ion Trap mass spectrometer (Thermo Scientific, Germany). Each full MS (m/z range of 300 to 2,000) scan was followed by three MS/MS scans of the most abundant precursor ions in the MS spectra. For protein identification, MS/MS spectra were analyzed by MASCOT (Matrix Science, http://www.matrix science.com). The genome sequence database of *N. pentaromativorans* US6-1 (GI:359402640) was downloaded from NCBI and used for protein identification. Mass tolerance of the parent or fragment ion was 0.8 Da. Cabamidomethylation of cysteine and oxidation of methionine were considered to be variable modifications of tryptic peptides in the MS/MS analysis.

### Nano-UPLC-MS^E^ Tandem Mass Spectrometry and database search

An alternative MS/MS analysis was conducted using a nano-ACQUITY Ultra Performance LC Chromatography™ equipped Synapt™ HDMS System (Waters Corporation, MA, USA) as described previously [17]. The flow-through peptides were applied to a nano-ACQUITY UPLC BEH300 C18 RP column (180 µm ×250 mm, particle size, 1.7 µm). Trypsin-digested peptide mixtures were loaded onto the enrichment column (180 µm ×20 mm, particle size, 5 µm), which was equilibrated with mobile phase A (3% acetonitrile in water with 0.1% formic acid) to remove salts and concentrate the peptides. Flow-through peptides were directly applied to a nano-ACQUITY UPLC BEH300 C18 RP column (180 µm ×250 mm, particle size, 1.7 µm) at a flow rate of 300 nl/min. The step gradient was as follows: 3−40% mobile phase B (97% acetonitrile in water with 0.1% formic acid) for 95 min, 40−70% mobile phase B for 20 min, and then a sharp increase to 80% mobile phase B for 10 min. MS data analysis was carried out with the continuum LC-MS^E^ data using ProteinLynx GlobalServer (PLGS) version 2.3.3 (Waters Corporation). The criteria for protein identification used in the PLGS search engine were applied with a peptide tolerance of 100 ppm, a fragment tolerance of 0.2 Da, and a missed cleavage allowance of 1. Analysis of quantitative changes in protein abundance (>95% confidence based on peptide ion peak intensities observed in low collision energy mode (MS) in a triplicate set) was conducted using Expression^™^ Software version 2(Waters Corporation).

### Cluster analysis of proteomic data and prediction of protein properties

The emPAI values were used in the cluster analysis of all analyzed proteins, and the proteome dataset was z-transformed and median-centered normalized. Analysis of the proteome dataset was performed by Pearson correlation and average linkage hierarchical clustering by Cluster and TreeView [18]. All proteins identified by proteomic analysis were classified according to the cluster of orthologous groups (COG) functions, and their subcellular localization of the identified proteins was predicted by Cello (v. 2.0; http://cello.life.nctu.edu.tw/) [19]. Prediction of trans-membrane topology was performed using Phobius (http://phobius.sbc.su.se/) [20].

### Purification and characterization of plasmid pLA1

*N. pentaromativorans* US6-1 was grown in LB broth containing 10 g/L NaCl. Plasmid DNA was isolated using a standard alkaline lysis procedure, and purified on NucleoBond columns (NucleoBond Plasmid BAC Maxi kit, Clontech, USA). Genomic DNA was shared by careful pipetting of the DNA solution up and down several times with a 200 µl Pipetman tip. Purified plasmid DNA was analyzed in a 1% (w/v) agarose gel by clamped homogeneous electrical field (CHEF) gel electrophoresis (Bio-Rad, USA). CHEF parameters were set according to the manufacturer's protocol, including a circulating temperature of 14°C, electric current of 350 mA, and a pulse time of 35 s for 28 h. After electrophoresis, the gel was stained with ethidium bromide solution for 30 min, washed with TBE for 30 min, and DNA bands were detected using the Gel-Doc System (Bio-Rad, USA).

## Results

### Comparative analysis of dioxygenase enzymes in *Novosphingobium pentaromativorans* US6-1 in response to polycyclic and monocyclic aromatic hydrocarbons

**Figure 1:**
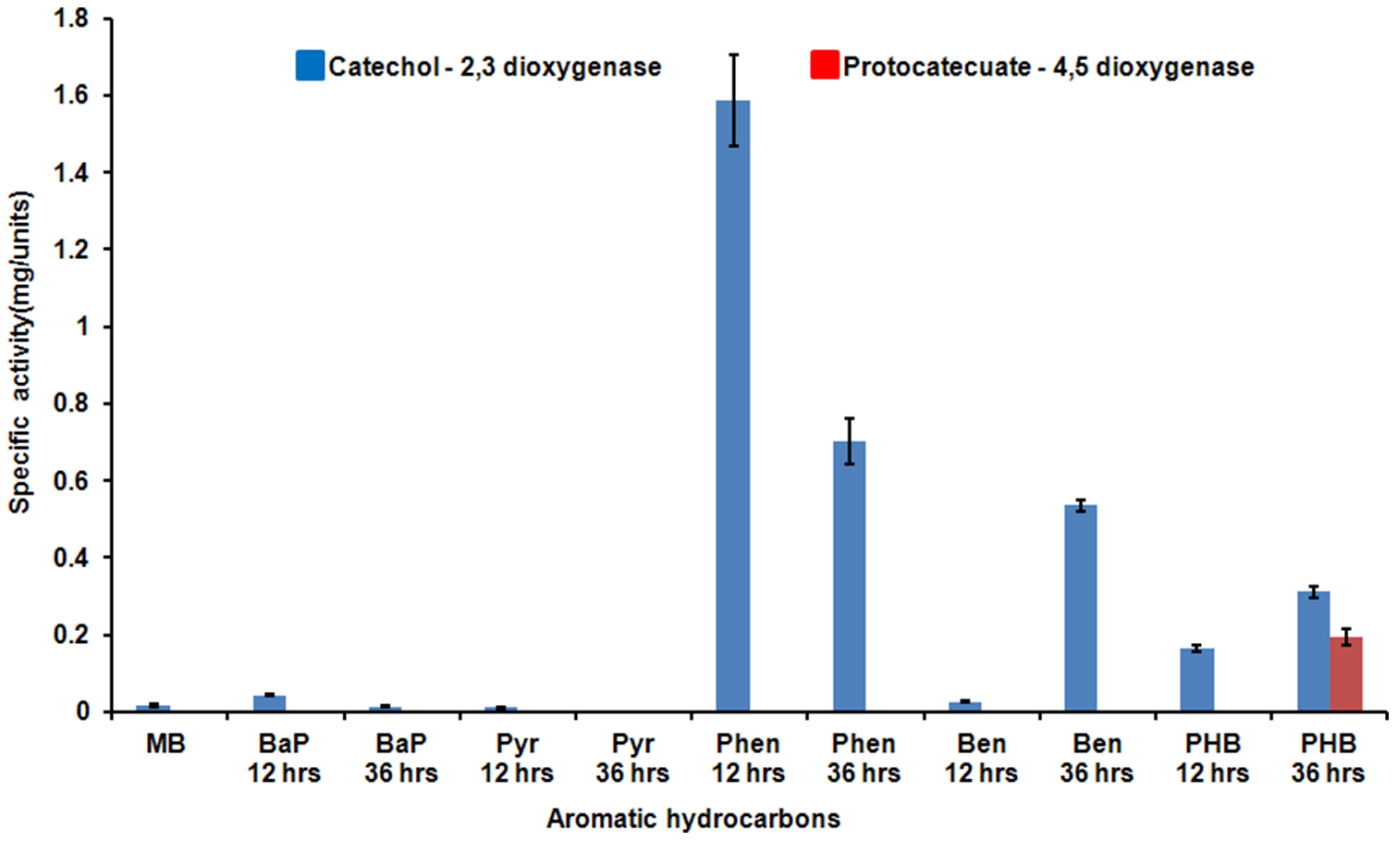
Induction of two extradiol dioxygenases (catechol 2,3-dioxygenase and protocatechuate 4,5-dioxygenase) in polycyclic aromatic hydrocarbon or monocyclic aromatic hydrocarbon cultivation. MB; Marine broth, BaP; Benzo(a)pyren, Pyr; Pyren, Phen; Phenanthrene, Ben; Benzoate, PHB; *p*-Hydroxybenzoate.

*N. pentaromativorans* US6-1 were pre-cultured in MB to obtain sufficient biomass to determine dioxygenase activity and proteomic analysis. Approximately equal quantities of cells were transferred into PAH and MAH culture media. Although all culture has same cell mass, each has different OD_600_ values because of different absorbance of PAHs and MAHs. OD values are as follow; benzo(a)pyren (0.5146 at OD_600_), pyren (0.5612 at OD_600_), phenanthren (0.604 at OD_600_), benzoate (0.4242 at OD_600_), and *p*- hydroxybenzoate (0.4366 at OD_600_). After 12 h incubation, delta OD values of benzo(a)pyren, pyren, phenanthren, benzoate, and *p*-hydroxybenzoate were +0.0101, +0.1461, +0.0628, −0.0347, −0.0199, respectively. After 36 h incubation, delta OD values of benzo(a)pyren, pyren, phenanthren, benzoate, and *p*-hydroxybenzoate were +0.0013, +0.0254, −0.008, −0.0492, +0.5695, respectively. The bacteria were harvested after 12 h and 36 h incubation for enzyme activity assay and proteome analysis. Highest delta OD values of benzo(a)pyren and pyren were +0.0101 and +0.1461 at 12 hours incubation, respectively. Phenanthren was continually increased until 48 hours (+0.0628). Unexpectedly, cell mass under benzoate culture condition was not increased, whereas degrading enzyme activities were significantly increased. The activities of four major dioxygenases (CD1,2, CD2,3, PCD3,4 and PCD4,5) were assayed using protein extracts from *N. pentaromativorans* US6-1 cultured in the presence of three PAHs and two MAHs to determine which biodegradation pathways were induced. Analysis of the *N. pentaromativorans* US6-1 genome indicated the presence of only extradiol oxygenase genes; however, many unspecified oxygenase genes were identified. In attempting to determine if *N. pentaromativorans* US6-1 also expressed intradiol oxygenase activities, we selected the four dioxygenase enzymes (CD1,2, CD2,3, PCD3,4 and PCD4,5) that we considered would cover most degradation pathways in aerobic cultures. No activity of the intradiol dioxygenases CD1,2 and PCD3,4 was detected. Activity of CD2,3 was high in cells cultured in media containing phenanthrene (1.59 U/mg at 12 h cultivation), benzoate (0.53 U/mg at 36 h cultivation), and *p*-hydroxybenzoate (0.31 U/mg at 36 hr cultivation), whereas, CD2,3 activity was detected to some degree in cells cultured under all conditions (0.02−0.04 U/mg), including MB media (Fig. 1). Activity of PCD4,5 was only detected in cells cultured in *p*-hydroxybenzoate (0.20 U/mg at 36 hr cultivation) media (Fig. 1). This suggests that the CD2,3 pathway could be a primary pathway for the degradation of PAHs and benzoate, while *p*-hydroxybenzoate is broken-down via the PCD4,5 or CD2,3 pathway in *N. pentaromativorans* US6-1.

### Proteomic analysis of *Novosphingobium pentaromativorans* US6-1 cultured with polycyclic aromatic hydrocarbons

**Figure 2:**
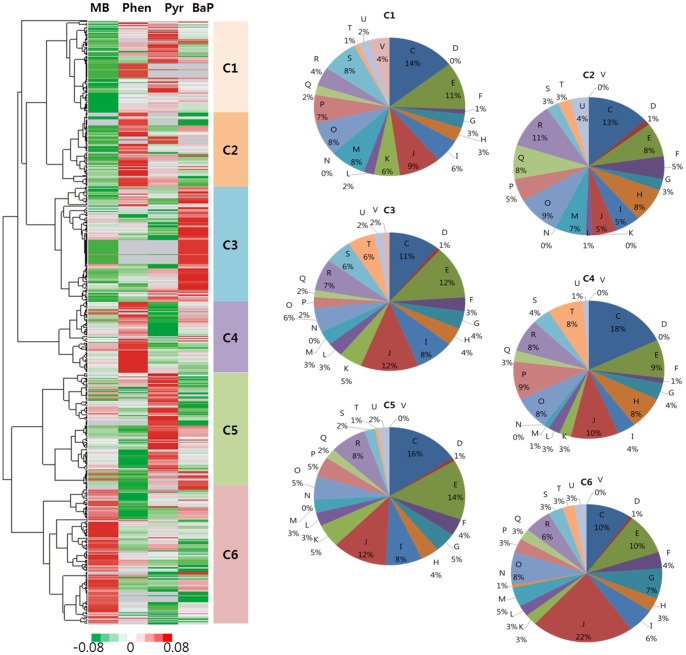
Cluster analysis of the proteomes of *N. pentaromativorans* US6-1 induced in polycyclic aromatic hydrocarbons. Proteome datasets were obtained from stain US6-1 cultured from four conditions (MB; Marine broth, BaP; Benzo(a)pyren, Pyr; Pyren, Phen; Phenanthrene) and were hierarchically clustered. Up-regulated or down-regulated proteins were indicated as red or green, respectively. Identified proteins were categorized into six groups (C1∼C6) according to differential induction in PAHs. Each group was divided by COG function (Table S2). Subgroup C ∼ V in COG function were defined as follows; [C] Energy production and conversion, [D] Cell cycle control, cell division, chromosome partitioning, [E] Amino acid transport and metabolism, [F] Nucleotide transport and metabolism, [G] Carbohydrate transport and metabolism, [H] Coenzyme transport and metabolism, [I] Lipid transport and metabolism, [J] Translation, ribosomal structure and biogenesis, [K] Transcription, [L] Replication, recombination and repair, [M] Cell wall/membrane/envelope biogenesis, [N] Cell motility, [O] Posttranslational modification, protein turnover, chaperones, [P] Inorganic ion transport and metabolism, [Q] Secondary metabolites biosynthesis, transport and catabolism, [R] General function prediction only, [S] Function unknown, [T] Signal transduction mechanisms, [U] Intracellular trafficking, secretion, and vesicular transport, [V] Defense mechanisms.

Cytosolic proteins were prepared for shotgun proteomics using 1-DE/LC MS/MS from cells cultured in different PAHs. Approximately 650−718 proteins were identified in cells grown in the presence of phenanthrene, pyrene, and benzo(a)pyrene. Comparative analysis between the three PAHs was made with cells grown in MB as a control (Table S1 and Fig. S1). Analysis revealed that 494 proteins were commonly induced by all culture media, with 26−32 unique to the different aromatic hydrocarbons used as sole carbon sources. The identified proteins were divided into six groups (C1−C6) according to cluster analysis with each protein group arranged according to COG functions (Fig 2). Enzymes of the PAH and MAH catabolic pathways were clustered in group C2 and group C6, and were expressed at higher levels in the presence of phenanthrene. This was particularly noticeable for the degradation enzymes associated with ‘secondary metabolite biosynthesis, transport and catabolism [Q]’ (Table S2). Proteins in group C6 were strongly induced in those cells grown in MB and the primary up-regulated COG protein group was ‘Translation, ribosomal structure and biogenesis [J]’. The ribosomal proteins induced during growth in MB increased by more than 1.69-fold compared to PAHs. ‘Proteins uncharacterized or unknown [R or S]’ were relatively higher in C3 group proteins, which were abundant under benzo[a]pyrene culture conditions.

A notable outcome of the proteomic analysis was the strong induction of genes originating from pLA1. This large plasmid encodes 199 genes, and approximately 27 are clustered, considered to be a putative coding region for LMW aromatic-hydrocarbon degradation. These genes could regulate biodegradation of bicyclic aromatic ring compounds through to tricarboxylic acid cycle metabolites, such as acetaldehyde. Approximately 27−36 proteins coded by genes located on pLA1 were induced by three PAHs. Among the putative biodegradation genes, 24, 19, and 12 proteins were up-regulated in the presence of phenanthrene, pyrene, and benzo(a)pyrene, respectively, compared to MB (Table 1). Comparative results showed that the greatest amount of protein induction occurred in the presence of phenanthrene, which is consistent with the results of the dioxygenase activity assay (Fig. 1). Enzymes catalyzing the breakdown of bicyclic aromatic compounds to acetyl CoA were strongly induced. Ring-hydroxylating dioxygenase, dihydrodiol dehydrogenase, putative biphenyl-2,3-diol-1,2-dioxygenase, and catechol 2,3-dixoygenase were included. Although the activity of catechol 2,3-dixoygenase in those cultures containing pyrene was low, proteomic results showed a strong induction of both this enzyme and others involved in related biodegradation steps (Table 1). The signal intensity for those enzymes produced by plasmid pLA1 in the presence of benzo(a)pyrene was lower than that for the other PAHs phenanthrene and pyrene.

**Table 1:** Induction of biodegradation genes of plasmid pLA1 according to different polycyclic aromatic hydrocarbons (PAHs).

Accesions No.	Description Name	Gene name	Gene locos	TM^a^	Subcellular location^b^	Relative concentration (Log2 ratio)^c^		
						BaP/MB	Phen/MB	Pyr/MB
gi|359402453	AcrB/AcrD/AcrF family protein	-	NSU_pLA1101	10	InnerMembrane	MB	MB	MB
gi|359402457	transposase of IS6100	-	NSU_pLA1105	0	Cytoplasmic	MB	-	MB
gi|359402458	regulator	-	NSU_pLA1106	0	OuterMembrane	-	Phen	-
gi|359402463	Semi aldehyde dehydrogenase	-	NSU_pLA1111	0	Cytoplasmic	0.104	0.376	0.056
gi|359402464	tonB-dependent receptor	-	NSU_pLA1112	1	OuterMembrane	-0.073	0.139	-0.060
gi|359402465	putative 2-hydroxychromene-2-carboxylate isomerase	nahD	NSU_pLA1113	0	Cytoplasmic	0.084	0.298	0.119
gi|359402466	putative large subunit of oxygenase	ahdA1c	NSU_pLA1114	0	Cytoplasmic	−0.068	0.323	0.092
gi|359402467	putative small subunit of oxygenase	ahdA2c	NSU_pLA1115	0	Cytoplasmic	−0.225	0.302	−0.074
gi|359402469	putative biphenyl-2,3-diol 1,2-dioxygenase	bphC	NSU_pLA1117	0	Cytoplasmic	0.150	0.345	0.065
gi|359402470	putative large subunit of toluene/benzoate dioxygenase	xylX	NSU_pLA1118	0	Cytoplasmic	MB	0.487	-
gi|359402471	putative small subunit of toluene/benzoate dioxygenase	xylY	NSU_pLA1119	0	Cytoplasmic	0.026	0.399	0.149
gi|359402472	4-hydroxythreonine-4-phosphate dehydrogenase	-	NSU_pLA1120	0	Cytoplasmic	−0.274	0.722	0.252
gi|359402473	salicylate 1-hydroxylase alpha subunit	bphA1d	NSU_pLA1121	0	Cytoplasmic	−0.066	0.624	0.032
gi|359402474	salicylate 1-hydroxylase beta subunit	bphA2d	NSU_pLA1122	0	Cytoplasmic	MB	0.442	0.228
gi|359402475	glutathione S-Transferase	bphK	NSU_pLA1123	0	Periplasmic	−0.186	0.511	−0.023
gi|359402476	putative 2-hydroxymuconic semialdehyde hydrolase	xylF	NSU_pLA1124	0	Cytoplasmic	-	Phen	-
gi|359402477	catechol 2,3-dioxygenase	xylE	NSU_pLA1125	0	Cytoplasmic	0.062	0.689	0.562
gi|359402479	2-hydroxymuconic semialdehyde dehydrogenase	xylG	NSU_pLA1127	0	Cytoplasmic	0.032	0.457	-
gi|359402480	putative 2-hydroxypent-2,4-dienoate hydratase	xylJ	NSU_pLA1128	0	Cytoplasmic	0.166	0.519	0.215
gi|359402481	acetaldehyde dehydrogenase	xylQ	NSU_pLA1129	0	Cytoplasmic	0.112	0.682	0.398
gi|359402482	4-hydroxy-2-oxovalerate aldolase	phnJ	NSU_pLA1130	0	Cytoplasmic	0.084	0.513	0.759
gi|359402483	4-oxolocrotonate decarboxylase	phnK	NSU_pLA1131	0	Cytoplasmic	−0.165	0.445	0.302
gi|359402486	salicylaldehyde dehydrogenase	-	NSU_pLA1134	0	Cytoplasmic	−0.141	0.248	−0.020
gi|359402487	dihydrodiol dehydrogenase	-	NSU_pLA1135	0	Cytoplasmic	−0.150	0.541	0.136
gi|359402488	hypothetical protein NSU_pLA1136	-	NSU_pLA1136	0	Cytoplasmic	MB	0.313	0.087
gi|359402490	ferredoxin reductase component of dioxygenase	ahdA4	NSU_pLA1138	0	Cytoplasmic	0.207	0.395	0.247
gi|359402491	putative cis-(methyl)benzoate dihydrodiol dehydrogenase	xylL	NSU_pLA1139	0	Cytoplasmic	MB	MB	MB
gi|359402492	putative 2-hydroxy-benzylpyruvate aldolase	nahE	NSU_pLA1140	0	Periplasmic	0.154	0.744	0.268
gi|359402495	hypothetical protein NSU_pLA1143	-	NSU_pLA1143	0	Cytoplasmic	-	Phen	-
gi|359402507	ring hydroxylating dioxygenase alpha subunit	phnA1a	NSU_pLA1155	0	Extracellular	0.039	0.784	0.166
gi|359402509	ring hydroxylating dioxygenase beta subunit	phnA2a	NSU_pLA1157	0	Cytoplasmic	−0.033	0.939	0.420
gi|359402511	hypothetical protein NSU_pLA1159	-	NSU_pLA1159	0	Cytoplasmic	0.182	0.839	0.528
gi|359402512	putative reductase	-	NSU_pLA1160	0	Cytoplasmic	0.112	1.004	0.509
gi|359402513	3-isopropylmalate dehydrogenase	-	NSU_pLA1161	0	Cytoplasmic	BaP	Phen	Pyr
gi|359402514	hypothetical protein NSU_pLA1162	-	NSU_pLA1162	0	Periplasmic	BaP	Phen	Pyr
gi|359402516	hypothetical protein NSU_pLA1164	-	NSU_pLA1164	0	Cytoplasmic	-	Phen	-
gi|359402531	acid phosphatase	-	NSU_pLA1179	0	Periplasmic	-	Phen	-
gi|359402532	RND efflux system, outer membrane lipoprotein, NodT family	cmeC	NSU_pLA1180	0	OuterMembrane	-	-	Pyr
gi|359402533	acriflavin resistance protein B	cmeB	NSU_pLA1181	11	InnerMembrane	-	Phen	Pyr
gi|359402534	secretion protein HlyD	cmeA	NSU_pLA1182	0	OuterMembrane	BaP	Phen	Pyr

a Prediction of cellular location by CELLO v.2.5: http://cello.life.nctu.edu.tw/.

b Prediction of the no. of transmembrane regions by TMHMM Server v. 2.0: http://www.cbs.dtu.dk/services/TMHMM/.

c Log2 ratio calculated according to emPAI values.

Abbreviations MB: marine broth, BaP: Benzo(a)pyren, Phen: phenanthrene, Pyr: pyren.

These results were verified by proteomic analysis using Nano-UPLC MS, confirming the up-regulation of *N. pentaromativorans* US6-1 biodegradation genes by phenanthrene, pyrene, and benzo(a)pyrene (Table 2). These results showed that the biodegradation genes located on pLA1 play a major role in the utilization of PAHs as sole carbon sources in *N. pentaromativorans* US6-1.

**Table 2:** Induction analysis of biodegradation genes of plasmid pLA1 according to polycyclic aromatic hydrocarbons (PAHs) by Nano-UPLC-MS^E^ (MS^E^ method).

Accesions. No	Description name	Gene name	Gene locos	Gene location	Subcellular location	TM	BaP/MB	Phen/MB	Pyr/MB
gi|359402463	semialdehyde dehydrogenase	xylQ	NSU_pLA1111	pLA1	Cytoplasmic	0	-	-	Pyrene
gi|359402464	tonB-dependent receptor	ORF88	NSU_pLA1112	pLA1	OuterMembrane	1	MB	1.34	0.41
gi|359402469	putative biphenyl-2,3-diol 1,2-dioxygenase	bphC	NSU_pLA1117	pLA1	Cytoplasmic	0	1.43	3.71	2.01
gi|359402473	salicylate 1-hydroxylase alpha subunit	bphA1d	NSU_pLA1121	pLA1	Cytoplasmic	0	MB	3.1	MB
gi|359402474	salicylate 1-hydroxylase beta subunit	bphA2d	NSU_pLA1122	pLA1	Cytoplasmic	0	-	Phen	-
gi|359402475	glutathione S-Transferase	bphK	NSU_pLA1123	pLA1	Periplasmic	0	0.65	3.6	1.16
gi|359402479	2-hydroxymuconic semialdehyde dehydrogenase	xylG	NSU_pLA1127	pLA1	Cytoplasmic	0	-	Phen	Pyrene
gi|359402486	salicylaldehyde dehydrogenase	nahF	NSU_pLA1134	pLA1	Cytoplasmic	0	0.77	2.36	1.38
gi|359402492	putative 2-hydroxy-benzylpyruvate aldolase	nahE	NSU_pLA1140	pLA1	Periplasmic	0	Benzo	Phen	Pyrene
gi|359402507	ring hydroxylating dioxygenase alpha subunit	phnA1a	NSU_pLA1155	pLA1	Extracellular	0	MB	5.21	1.54
gi|359402509	ring hydroxylating dioxygenase beta subunit	phnA2a	NSU_pLA1157	pLA1	Cytoplasmic	0	-	Phen	-
gi|359402511	hypothetical protein NSU_pLA1159	-	NSU_pLA1159	pLA1	Cytoplasmic	0	1.1	7.17	1.86
gi|359402512	putative reductase	-	NSU_pLA1160	pLA1	Cytoplasmic	0	-	Phen	-

Abbreviations MB: marine broth, BaP: Benzo(a)pyren, Phen: phenanthrene, Pyr: pyren.

### Proteomic analysis of *Novosphingobium pentaromativorans* US6-1 cultured with monocyclic aromatic hydrocarbons

**Figure 3:**
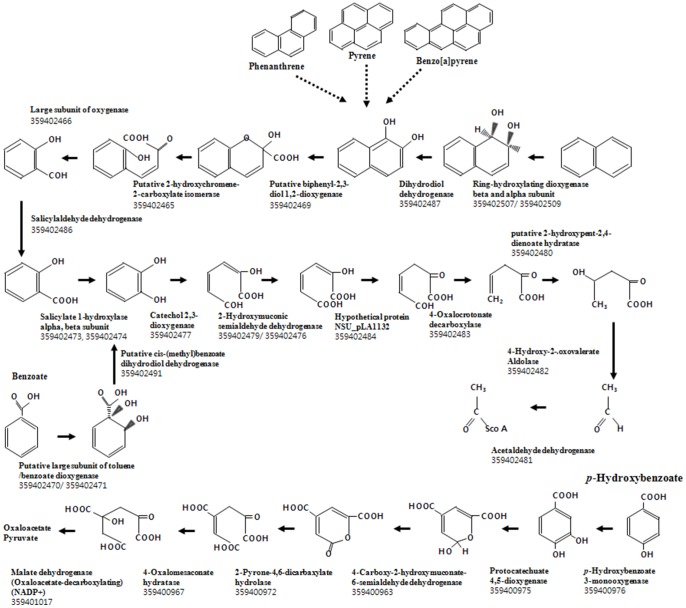
Two extradiol biodegradation pathways of *N. pentaromativorans* US6-1. Two biodegradation pathways were identified by genomic analysis using KEGG program and the induced enzymes were confirmed by proteomic analysis of *N. pentaromativorans* US6-1. PAHs are assumed to converge into 1,2-dihydroxynapthalene, whereas, ring-hydroxylating dioxygenase and dihydrodiol dehydrogenase are induced in our PAHs culture conditions. Up: Catechol 2,3-dioxygenase pathway (large plasmid pLA1). Down: Protocatechuate 4,5-dioxygenase pathway (chromosome).

In response to the differential induction of extradiol dioxygenases of *N. pentaromativorans* US6-1 in the presence of benzoate and *p*-hydroxybenzoate, a comparative proteome analysis was conducted. Approximately 1,475 proteins were identified. 126 were induced by benzoate, and 85 by *p*-hydroxybenzoate (Table S1 and Fig. S1.). Genomic analysis revealed the presence of a CD2,3 gene and a ring-hydroxylating gene on pLA1, which are responsible for the initial step of the lower catabolic pathway of aromatic hydrocarbons. Notably, four PCD4,5 genes (small and large subunits) were found to be localized on the chromosome. Proteome analysis showed that when *N. pentaromativorans* US6-1 was cultured in *p-*hydroxybenzoate, two PCD4,5 and *p*-hydroxybenzoate degradation enzymes were induced (Table 3 and Fig. 3). These genes (gene number NSU_3623−NSU_3811) were clustered on contig 58 of the chromosome (Table S3). However, none of the three PAHs or benzoate induced the expression of PCD4,5 and *p*-hydroxybenzoate degradation enzymes, suggesting that the chromosomal-born protocatechuate pathway plays a role in the breakdown of *p*-hydroxybenzoate, but not in the degradation of PAHs or benzoate. Unexpectedly, the biodegradation genes on pLA1 were expressed in response to *p*-hydroxybenzoate, despite having no direct role in the breakdown of this compound.

**Table 3:** Induction of two extradiol biodegradation pathways genes by in *N. pentaromativorans* US6-1 cultured in *p*-hydroxybenzoate.

Accesions No.	Description name	Gene name	Gene locus	Gene Location	TM	Subcellular location	Ben	PHB
gi|359402477	catechol 2,3-dioxygenase	xylE	NSU_pLA1125	pLA1	0	Cytoplasmic	0.074	0.217
gi|359402479	2-hydroxymuconic semialdehyde dehydrogenase	xylG	NSU_pLA1127	pLA1	0	Cytoplasmic	0.162	0.207
gi|359402480	putative 2-hydroxypent-2,4-dienoate hydratase	xylJ	NSU_pLA1128	pLA1	0	Cytoplasmic	0.137	0.099
gi|359402482	4-hydroxy-2-oxovalerate aldolase	phnJ	NSU_pLA1130	pLA1	0	Cytoplasmic	0.263	0.437
gi|359402483	4-oxolocrotonate decarboxylase	phnK	NSU_pLA1131	pLA1	0	Cytoplasmic	0.072	0.126
gi|359402484	hypothetical protein NSU_pLA1132	-	NSU_pLA1132	pLA1	0	Cytoplasmic	-	0.069
gi|359400963	4-carboxy-2-hydroxymuconate-6-semialdehyde dehydrogenase	-	NSU_3623	Chr.	0	Cytoplasmic	0.025	1.061
gi|359400964	protocatechuate 4,5-dioxygenase, beta chain	-	NSU_3624	Chr.	0	Cytoplasmic	-	0.336
gi|359400965	protocatechuate 4,5-dioxygenase, alpha chain	-	NSU_3625	Chr.	0	Periplasmic	-	0.354
gi|359400967	4-oxalmesaconate hydratase	-	NSU_3627	Chr.	0	Cytoplasmic	-	0.127
gi|359400972	2-pyrone-4,6-dicarbaxylate hydrolase	-	NSU_3632	Chr.	0	Periplasmic	-	0.067
gi|359400974	protocatechuate 4,5-dioxygenase, beta chain	-	NSU_3634	Chr.	0	Cytoplasmic	0.021	0.102
gi|359400975	protocatechuate 4,5-dioxygenase, alpha chain	-	NSU_3635	Chr.	0	Periplasmic	-	0.013
gi|359400976	p-hydroxybenzoate 3-monooxygenase	-	NSU_3636	Chr.	0	Cytoplasmic	0.097	1.579
gi|359401017	malate dehydrogenase (oxaloacetate-decarboxylating)(NADP+)	-	NSU_3677	Chr.	1	Cytoplasmic	0.035	0.255

Abbreviations: Ben; Benzoate, PHB; *p*-Hydroxybenzoate, Chr; chromosome, pLA1; large plasmid.

## Discussion

The genomic sequence of the bacterium *N. pentaromativorans* US6-1, which is able to utilize PAHs as its sole carbon source, was reported recently [10], [11]. However, the genes coding proteins that are important for the biodegradation of PAH have not been completely annotated, and until now, their function has remained largely speculative. Two putative clusters containing the genes necessary for the breakdown of aromatic compounds were detected in the biodegradation gene region of plasmid pLA1 and contig 58 of the chromosome (Table S3). In this study, a proteomic analysis of *N. pentaromativorans* US6-1 cultured in the presence of three different PAHs was conducted to examine the differential expression of biodegradative genes. The results indicate that PAHs strongly induced the expression of biodegradation genes located on plasmid pLA1 (Fig 2 and Table 1), but that other biodegradation gene clusters on contig 58 were not induced. These results suggest that the biodegradation genes on plasmid pLA1 are essential for the biodegradation of PAHs. Semi-quantitative proteomic analysis using emPAI revealed that tri- (phenanthrene), tetra- (pyrene), and penta-aromatic compounds (benzo(a)pyrene) induce a differential biodegradation capacity (Table 1 and 2). The total amount of biodegrading enzymes induced in the presence of phenanthrene was estimated to be nine times greater than that of benzo(a)pyrene (Table 1). However, we found a discrepancy between enzyme activity and the amount of CD2,3 expression induced in cells cultured in the presence of pyrene, and the reason for the instability of CD2,3 induced by pyrene-containing culture media remains unclear. Because genes that regulate the biodegradation of bicyclic aromatic compounds were identified on pLA1, genes for high-molecular-weight PAHs should be found on the chromosome. An analysis using the program pFam revealed that several putative aromatic compound-degrading genes are scattered throughout the chromosome (contigs 58, 54, and 55), although they were not induced significantly under our culture conditions. We also considered the possibility that the cytochrome P450 monooxygenases (CYPs) may provide alternative biodegradation pathways. Proteomic analysis showed that four cytochrome P450 proteins (NSU_2269, 2261, 2259, and 2257) were present, with the hypothetical protein NSU_2261 assigned as a cytochrome P450 monooxygenases in uniprot Blast analysis. Induction level was very low, suggesting that CYP has no direct involvement with the biodegradation of PAHs (Table S1), and this was further supported by the genomic analysis of *N. pentaromativorans* US6-1. The degradation of PAHs was catalyzed by enzymes with broad substrate specificities. Consequently, further biochemical assay and overexpression are required to determine related biodegrading genes [21]–[23], coupled with a more accurate proteomic study and optimization of culture conditions to understand PAH metabolism in *N. pentaromativorans* US6-1. Plasmids similar to pLA1 have been identified in *Sphingomonas aromaticivorans* F199 and *Sphingomonas sp.* strain KA1 [24], [25]. *S. aromaticivorans* F199 contains a large plasmid, pNL1, which possesses 186 ORFs and 79 genes that mediate catabolism or transport of aromatic compounds, such as mono-aromatic compounds (m-xylene and p-cresol) and bicyclic aromatic compounds (biphenyl and naphthalene). Plasmid pCAR3 from *Sphingomonas sp.* strain KA1 contains a number of carbazole degradation genes. A comparative analysis of DNA sequence of pLA1 and pNL1 reveals that they are significantly homologous (more than 36%) and each of the ORFs in pLA1 has more than 61% amino acid sequence homology with its corresponding ORFs (Fig S2). These plasmids are categorized into four groups according to the repA proteins, with pLA1 from *N. pentaromativorans* US6-1 belonging to the Rep_3 superfamily.

**Figure 4:**
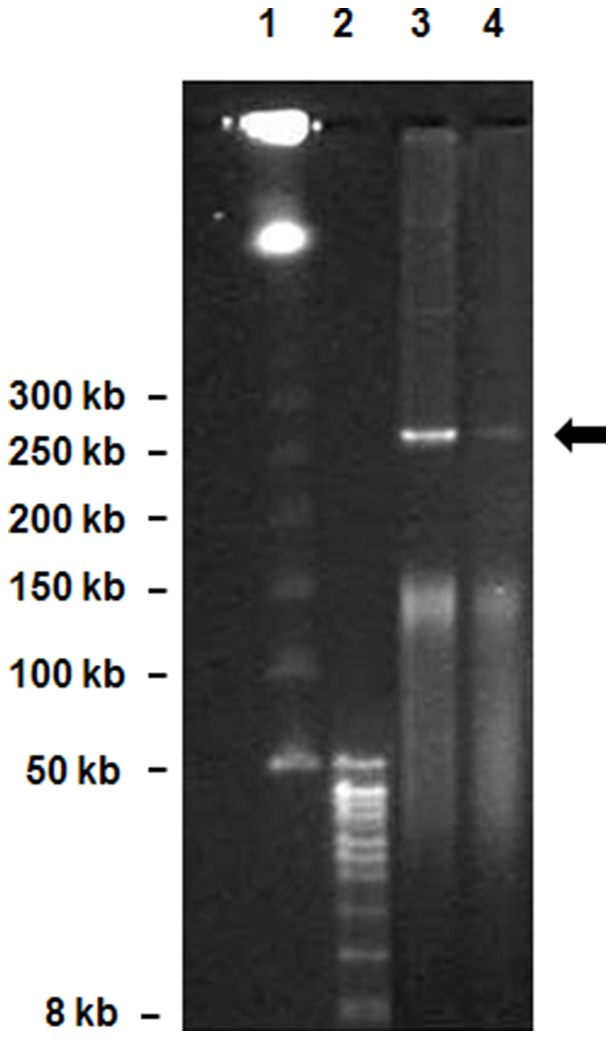
Purification of Plasmid pLA1 of *N. pentaromativorans* US6-1. Open circled plasmid was indicated by an arrow. Lane 1; ProMega-Markers Lambda Ladders, lane 2; DNA size standards 8∼48 kb, lane 3; plasmid from *N. pentaromativorans* US6-1 cultured in LB broth, lane 4; plasmid from *N. pentaromativorans* US6-1 cultured in *p*-hydroxybenzoate media.

The proteomic characterization in this study revealed that *N. pentaromativorans* US6-1 utilizes different pathways for the breakdown of the two MAHs. Benzoate was degraded via the CD2,3 pathway encoded by genes located on plasmid pLA1, whereas *p*-hydroxybenzoate was broken down by the PCD4,5 route. The lower induction of biodegradation enzymes coded by pLA1 in the presence of MAHs such as benzoate and *p*-hydroxybenzote suggests that their regulation was optimized by PAHs or their metabolites. This assumption should be confirmed by further studies. The genes on plasmid pLA1 have been confirmed only by genomic sequencing and gene assembly. In a previous study, the presence of plasmid pLA1 in *N. pentaromativorans* US6-1 was not confirmed due to difficulties in the purification of large plasmid (>200 kb). Here, we confirmed the presence of pLA1 in *N. pentaromativorans* US6-1 by CHEF gel electrophoresis, and identified an open-circled plasmid of approximately >250 kb (Fig. 4). The presence of biodegradation genes on the plasmid was confirmed by PCR using the purified DNA as the template (data not shown).

In conclusion, two extradiol degradation pathways mediated by genes located in the plasmid and chromosome were separately induced by PAHs and MAHs. The large plasmid pLA1 plays a pivotal role in the degradation of bicylic aromatic compounds to acetyl CoA in *N. pentaromativorans* US6-1.

## Supporting Information

Figure S1 **Total number of identified proteins of** ***N. pentaromativorans*** **US6-1 by LC-MS/MS analysis.** A & B; The proteomes induced in PAHs and MAHs were identified using *N. pentaromativorans* US6-1 genome database. Benzoate (Ben), *p*-hydroxybenzoate (PHB), phenanthrene (Phen), pyrene (Pyr), banzo(a)pyrene (BaP). C & D; the proteomes induced in PAHs and MAHs were identified using plasmid (pLA1) database of *N. pentaromativorans* US6-1.
(TIF)

Figure S2 **Comparative analysis of pLA1 of** ***N. pentaromativorans*** **US6-1 and pNL1 of** ***N. aromaticivorans pentaromativorans*** **F199.** Biodegradation genes were indicated with red boxes.
(TIF)

Table S1 **Total identified proteins by LC-MS/MS analysis.**
(XLSX)

Table S2 **Clustering of total identified proteins of** ***N. pentaromativorans*** **US6-1.**
(XLSX)

Table S3 **Putative biodegradation gene clusters of** ***N. pentaromativorans*** **US6-1 and their proteomic result according to PAHs and MAHs.**
(XLSX)
