# Supplementary figures and images for: Proteomic Characterization of Plasmid pLA1 for Biodegradation of Polycyclic Aromatic Hydrocarbons in the Marine Bacterium, Novosphingobium pentaromativorans US6-1
Source: PLoS One. 2014 Mar 7;9(3):e90812. doi: 10.1371/journal.pone.0090812 (PMC3946609; doi:10.1371/journal.pone.0090812)

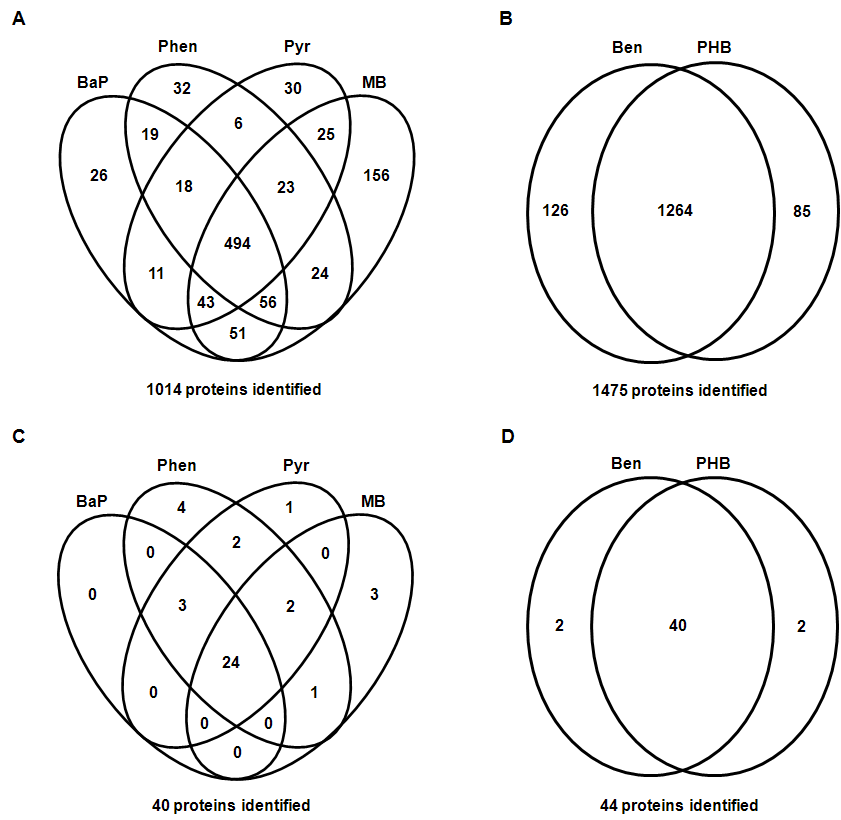

Supplement: Figure S1 — Total number of identified proteins of N. pentaromativorans US6-1 by LC-MS/MS analysis. A & B; The proteomes induced in PAHs and MAHs were identified using N. pentaromativorans US6-1 genome database. Benzoate (Ben), p-hydroxybenzoate (PHB), phenanthrene (Phen), pyrene (Pyr), banzo(a)pyrene (BaP). C & D; the proteomes induced in PAHs and MAHs were identified using plasmid (pLA1) database of N. pentaromativorans US6-1. (TIF) [file pone.0090812.s001.tif]

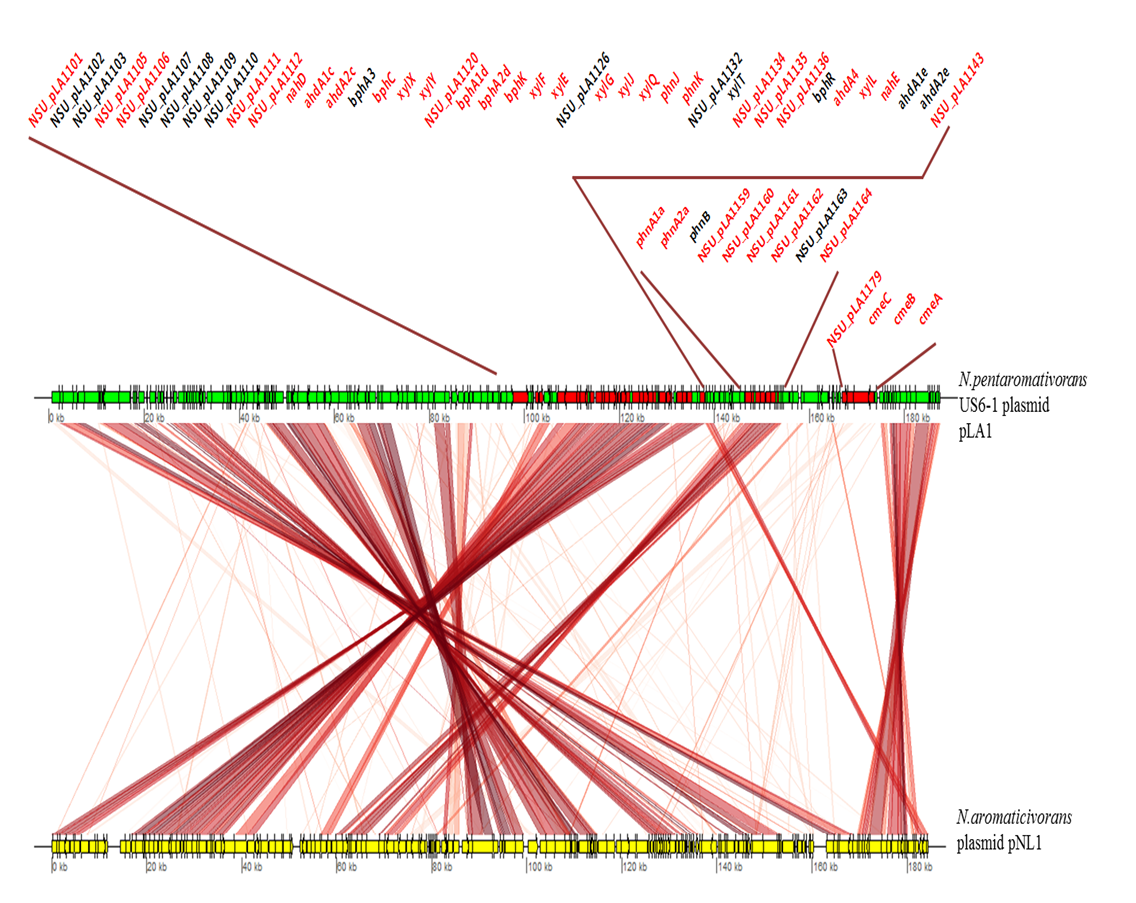

Supplement: Figure S2 — Comparative analysis of pLA1 of N. pentaromativorans US6-1 and pNL1 of N. aromaticivorans pentaromativorans F199. Biodegradation genes were indicated with red boxes. (TIF) [file pone.0090812.s002.tif]
